# Supplementary material for: Content-rich biological network constructed by mining PubMed abstracts
Source: BMC Bioinformatics. 2004 Oct 8;5:147. doi: 10.1186/1471-2105-5-147 (PMC528731; doi:10.1186/1471-2105-5-147)
Supplement: Additional File 2 — The original results of the above study (non-essential files are deleted to keep the file size under the limit set by BMC bioinformatics). [file 1471-2105-5-147-S2.bz2 › chilibotAdditionalFile2/dip30/198ID8652557E683/html/ERCC2_GTF2H1.html]

 


 **ERCC2** and **GTF2H1** 
  
Found 85 abstracts in PubMed, retrieved 30.  
 

 What does Google say? 
 PDF only 
| .edu only 

---

**Interactive relationship** (e.g. stimulation, inhibition, etc)

**Neutral relationship**- The xeroderma pigmentosum group D XPD  [ **ERCC2** ]  protein is a subunit of transcription factor TFIIH  [ **GTF2H1** ]  with DNA helicase activity.  Ref: 11709541 Hum Mol Genet, 2001
- XPD  [ **ERCC2** ]  is a component of the TFIIH  [ **GTF2H1** ]  transcription factor, which is essential for RNA polymerase II elongation.  Ref: 11334141 Antisense Nucleic Acid Drug Dev, 2001
- one of the roles of XPD  [ **ERCC2** ]  helicase is to anchor the cdk7 kinase to the core TFIIH  [ **GTF2H1** ] .  Ref: 11412842 FEBS Lett, 2001
- Using immunoprecipitates from cells coinfected with baculoviruses, we further validated the bridging function of XPD  [ **ERCC2** ] , which anchors CAK to the core TFIIH  [ **GTF2H1** ] .  Ref: 11445587 J Biol Chem, 2001
- As biochemists, we have characterized each component of TFIIH  [ **GTF2H1** ] , three of which are XPB and XPD  [ **ERCC2** ]  helicases and cdk7, a cyclin dependent kinase.  Ref: 11412842 FEBS Lett, 2001
- Cloning of a human homolog of the yeast nucleotide excision repair gene MMS19 and interaction with transcription repair factor TFIIH  [ **GTF2H1** ]  via the XPB and XPD  [ **ERCC2** ]  helicases.  Ref: 11071939 Nucleic Acids Res, 2000
- The XPD  [ **ERCC2** ]  gene encodes a subunit of the transcription factor II H TFIIH  [ **GTF2H1** ] , a complex involved in nucleotide excision repair NER and basal transcription.  Ref: 11737070 Clin Exp Immunol, 2001
- Trichothiodystrophy TTD is a rare hereditary multisystem disorder associated with defects in nucleotide excision repair NER as a consequence of mutations in XPD  [ **ERCC2** ] , XPB or TTDA, three genes that are all related to TFIIH  [ **GTF2H1** ] , the multiprotein complex involved in NER and transcription.  Ref: 12393803 Hum Mol Genet, 2002
- We also show that mutations in CSB, as well as in XPB and XPD  [ **ERCC2** ]  genes, all of which confer CS, disturb the RNA pol I TFIIH  [ **GTF2H1** ]  interaction within the CSB IP 150.  Ref: 12419226 Mol Cell, 2002
- The p44 subunit plays a crucial role in the overall activity of the transcription DNA repair factor TFIIH  [ **GTF2H1** ]  on the one hand its N terminal domain interacts with and regulates the XPD  [ **ERCC2** ]  helicase,.  Ref: 11319235 J Biol Chem, 2001
- Taken together, our results show that the p52 Tfb2 subunit of TFIIH  [ **GTF2H1** ]  regulates the function of XPB through pair wise interactions as described previously for p44 and XPD  [ **ERCC2** ] .  Ref: 12080057 J Biol Chem, 2002
- In both processes, TFIIH  [ **GTF2H1** ]  is implicated with local DNA unwinding, which is attributed to its helicase subunits XPB and XPD  [ **ERCC2** ] .  Ref: 11141066 Biochemistry, 2001
- These findings suggest that the nuclear entry of XPD  [ **ERCC2** ]  depends on its complexation with other proteins in the cytoplasm, possibly other components of the TFIIH  [ **GTF2H1** ]  complex.  Ref: 11266560 Nucleic Acids Res, 2001
- TFIIH  [ **GTF2H1** ]  is a multifunctional RNA polymerase II general initiation factor that includes two DNA helicases encoded by the Xeroderma pigmentosum complementation group B XPB and D XPD  [ **ERCC2** ]  genes and a cyclin dependent protein kinase encoded by the CDK7 gene.  Ref: 11331764 Proc Natl Acad Sci U S A, 2001
- General transcription factor IIH TFIIH  [ **GTF2H1** ]  consists of nine subunits cyclin dependent kinase 7 Cdk7, cyclin H and MAT1 forming the Cdk activating kinase or CAK complex, the two helicases Xpb Hay and Xpd  [ **ERCC2** ] , and p34, p44, p52 and p62 refs 1 3.  Ref: 12853965 Nature, 2003
- XPD  [ **ERCC2** ]  mutations prevent TFIIH  [ **GTF2H1** ]  dependent transactivation by nuclear receptors and phosphorylation of RARalpha.  Ref: 11955452 Cell, 2002
- Moreover, when XPD  [ **ERCC2** ]  mutations prevent interaction with the p44 subunit of TFIIH  [ **GTF2H1** ] , transactivation directed by certain nuclear receptors is inhibited, regardless of TTD versus XP phenotype, thus explaining the overlapping symptoms.  Ref: 12820975 Mol Cell, 2003

**Non-interactive relationship** (e.g. studied together, co-existance, homology, etc.)

- Transcriptional regulation of the TFIIH  [ **GTF2H1** ]  transcription repair components XPB and XPD  [ **ERCC2** ]  by the hepatitis B virus x protein in liver cells and transgenic liver tissue.  Ref: 11278765 J Biol Chem, 2001
- Regulation of cisplatin resistance and homologous recombinational repair by the TFIIH  [ **GTF2H1** ]  subunit XPD  [ **ERCC2** ] .  Ref: 12359753 Cancer Res, 2002
- In UV sensitive TTD, the TFIIH  [ **GTF2H1** ]  transcription factor containing XPB and XPD  [ **ERCC2** ]  helicase activities necessary for both transcription initiation and DNA repair is damaged.  Ref: 11369901 J Am Acad Dermatol, 2001
- Here we show that the Drosophila TFIIH  [ **GTF2H1** ]  component Xpd  [ **ERCC2** ]  negatively regulates the cell cycle function of Cdk7, the CAK activity.  Ref: 12853965 Nature, 2003
- In this report, we demonstrate an association of a C terminal domain of human RAD52 amino acids 302 418 with the XPB and XPD  [ **ERCC2** ]  subunits of transcription factor TFIIH  [ **GTF2H1** ]  and RNA polymerase II RNAPII.  Ref: 12372413 Biochem Biophys Res Commun, 2002
- Mutations in the XPD  [ **ERCC2** ]  helicase component of TFIIH  [ **GTF2H1** ]  can result in the diverse clinical features associated with xeroderma pigmentosum XP and trichothiodystrophy TTD.  Ref: 11734544 Hum Mol Genet, 2001
- Three polypeptides, primarily positioned 5 to the damage, are in close physical proximity to the psoralen lesion and thus are cross linked to the damaged DNA these proteins are RPA70, RPA32, and the XPD  [ **ERCC2** ]  subunit of TFIIH  [ **GTF2H1** ] .  Ref: 12138203 Mol Cell Biol, 2002
- This is an expected phenotype if the XPD  [ **ERCC2** ]  function is affected and indicates that the antisense approach may be an alternative in the study of TFIIH  [ **GTF2H1** ]  functions in Drosophila.  Ref: 11334141 Antisense Nucleic Acid Drug Dev, 2001
- We conclude that the severity of the clinical features in TTD patients and the clinical outcome of differentially mutated XPD  [ **ERCC2** ]  proteins is likely to depend both on the effects that each mutation has on the stability of TFIIH  [ **GTF2H1** ]  and on the transcriptional activity of the residual TFIIH  [ **GTF2H1** ]  complexes.  Ref: 12393803 Hum Mol Genet, 2002
- Mutations in XPB and XPD  [ **ERCC2** ]  TFIIH  [ **GTF2H1** ]  helicases have been related with three hereditary human disorders xeroderma pigmentosum, Cockayne syndrome, and trichothiodystrophy.  Ref: 12221129 Mol Biol Cell, 2002
- The XPD  [ **ERCC2** ]  protein performs not only an essential viability function as a structural component of transcription initiation factor TFIIH  [ **GTF2H1** ] , but also an NER function as a 5 to 3 DNA helicase within TFIIH  [ **GTF2H1** ]  that unwinds DNA on the 3 side of bulky lesions.  Ref: 11746743 Environ Mol Mutagen, 2001
- Co immunoprecipitation experiments revealed that hMMS19 directly interacts with the XPB and XPD  [ **ERCC2** ]  subunits of NER transcription factor TFIIH  [ **GTF2H1** ] .  Ref: 11071939 Nucleic Acids Res, 2000
- The antibody study indicated that they thus altered the conformation of one side of TFIIH  [ **GTF2H1** ] , consisting of p44, XPD  [ **ERCC2** ] , and Cdk activating kinase subunits, that is essential for the transition stage.  Ref: 12665589 Mol Cell Biol, 2003
- Inherited mutations of the TFIIH  [ **GTF2H1** ]  helicase subunits xeroderma pigmentosum XP B or XPD  [ **ERCC2** ]  yield overlapping DNA repair and transcription syndromes.  Ref: 11239393 Cell, 2001
- We report here the different ways in which four subunits of the basal transcription repair factor TFIIH  [ **GTF2H1** ]  XPB, XPD  [ **ERCC2** ] , p62 and p44 and the damage recognition XPC repair protein can enter the nucleus.  Ref: 11266560 Nucleic Acids Res, 2001
- It is not surprising that under these conditions some XPD  [ **ERCC2** ]  mutations affect interactions not only with p44, but also with MAT1, thus preventing either the CAK inhibitory function within CAK.XPD  [ **ERCC2** ]  and or the role of CAK within TFIIH  [ **GTF2H1** ]  and, consequently, explaining the variety of the XP phenotypes.  Ref: 11445587 J Biol Chem, 2001
- Herein we report that HBx represses two components of the transcription repair factor TFIIH  [ **GTF2H1** ] , XPB p89, and XPD  [ **ERCC2** ]  p80, both in p53 proficient and p53 deficient liver cells.  Ref: 11278765 J Biol Chem, 2001
- Inherited mutations in the XPD  [ **ERCC2** ]  subunit of the general transcription repair factor TFIIH  [ **GTF2H1** ]  yield the rare genetic disorder Xeroderma pigmentosum XP, the phenotypes of which cannot be explained solely on the basis of a DNA repair defect.  Ref: 11955452 Cell, 2002
- We have also measured TFIIH  [ **GTF2H1** ]  levels in cells in which different mutations in the XPD  [ **ERCC2** ]  gene are associated with clinical symptoms not of TTD but of the highly cancer prone disorder xeroderma pigmentosum XP.  Ref: 12393803 Hum Mol Genet, 2002
- We have recently completed screening of the National Cancer Institute human tumor cell line panel and demonstrated that among four nucleotide excision repair proteins XPA, XPB, XPD  [ **ERCC2** ] , and ERCC1, only the TFIIH  [ **GTF2H1** ]  subunit XPD  [ **ERCC2** ]  endogenous protein levels correlate with alkylating agent drug resistance.  Ref: 12359753 Cancer Res, 2002
- The xeroderma pigmentosum group D XPD  [ **ERCC2** ]  helicase subunit of TFIIH  [ **GTF2H1** ]  functions in DNA repair and transcription initiation.  Ref: 11242112 Nat Genet, 2001
